# Vision Screenings and Ophthalmology Referrals Among a Sample of Pediatricians in Florida

**DOI:** 10.7759/cureus.59473

**Published:** 2024-05-01

**Authors:** Meghan Sharma, Laura Huertas, Eleonore Savatovsky, Alana Grajewski

**Affiliations:** 1 Department of Ophthalmology, University of Miami Bascom Palmer Eye Institute, Miami, USA

**Keywords:** vision and eye screening, prevention ophthalmology, prevention science, primary screening, ophthalmology referrals, blindness prevention, vision screening

## Abstract

Introduction

In Florida, mandated school vision screenings begin around the age of five.^ ^However, a joint statement in the ophthalmology community recommends that primary care providers, including pediatricians, screen for eye and vision symptoms and signs starting at birth. This suggests that pediatricians may be the first to catch signs of early vision loss and refer pediatric patients to an ophthalmologist. This study aims to understand the current vision screening practices of a sample of pediatricians in Florida and how comfort levels with vision screenings may impact ophthalmology referrals.

Methods

A survey with 36 questions was created by the authors of the study and sent to pediatricians through digital newsletters to the Florida Chapter of the American Academy of Pediatrics and pediatric departments at Florida universities. Descriptive statistics were gathered regarding the demographics of pediatricians surveyed, vision screening attitudes and practices, vision concerns and ophthalmology referrals from pediatric clinics, and the most common reasons for referral to an ophthalmologist. The Jonckheere-Terpstra nonparametric trend test was used to examine whether decreased comfort performing vision screening on a child was associated with lower rates of urgent referrals to an optometrist or ophthalmologist.

Results

Forty-six responses were collected. Seventy-eight percent of pediatricians reported performing early childhood vision screening (n=36). There was considerable variability in vision screening practices among the pediatricians studied, with only 66% beginning screenings from zero to two years of age (n=24). Fifty percent of respondents reported receiving no previous training on performing vision screening, and less than half of respondents reported feeling “somewhat comfortable” or “extremely comfortable” with performing the exam (n=22, 48%). The trend between decreased comfort performing pediatric vision screening and lower rates of urgent eye care referrals was approaching statistical significance (p=0.0705). The majority of urgent referrals were provided by respondents who were somewhat or extremely comfortable with screening (n=13, 65%).

Conclusion

From this sample of pediatricians in Florida, most respondents reported performing early childhood vision screening, but there was notable variability in the way screenings were performed among pediatricians. Moreover, many had never received training on performing the exam or did not feel comfortable performing them. Decreased comfort with vision screenings was almost significantly associated with decreased urgent referrals to an ophthalmologist. Future studies should examine whether increased training on vision screenings may help improve standardization of screening practices among pediatrics and comfort with vision screenings.

## Introduction

In 41 USA states, childhood vision screenings are mandated at schools starting at kindergarten, or around the age of five [1]. Nevertheless, ocular pathologies can occur before the age of five and can include congenital cataracts, congenital glaucoma, retinoblastoma, or amblyopia among others. Furthermore, refractive error, strabismus, and amblyopia affect between 5% and 10% of preschool-age children [2]. Vision problems such as these have been shown to affect future academic performance and the social and mental health of children [3]. Early vision screening can help prevent vision impairment, which may be prevented or treated when addressed early [2]. For children younger than five years old, the age at which most mandatory school vision screening begins, eye health is typically monitored by a child’s primary care provider (PCP) or pediatrician. Any child who is found to have an ocular abnormality upon examination should be referred to a pediatric ophthalmologist for further evaluation [4].

A 2022 joint statement of the American Academy of Ophthalmology (AAO) and American Association for Pediatric Ophthalmology and Strabismus (AAPOS) recommended that PCPs, including pediatricians, screen for eye and vision symptoms, starting with an examination of the fundus/red reflex at birth. According to these guidelines, infants and toddlers should also have their eyes examined at each well-child visit from one month to four years of age. Autorefraction may be electively performed from 12 months to three years of age. Once the child demonstrates cooperation with ocular examination around the age of three and a half to four, visual acuity should be assessed [5]. These screening methods suggest that pediatricians may be the first to catch signs of an eye disease, particularly during the early years of life, and refer the patient to an ophthalmologist. This study aims to 1) understand current vision screening practices of a sample of pediatricians, and 2) evaluate how comfort levels with vision screenings may impact ophthalmology referrals. The study was performed in Florida, a US state, with mandatory school vision screening beginning at five years old [6]. This article was previously presented as a meeting abstract at the AAPOS 48th Annual Meeting on April 1, 2023. This article was also previously posted as a preprint in August 2023.

## Materials and methods

A 36-question survey was formulated by the authors of this study using the University of Miami Qualtrics digital survey platform. Qualtrics is an online platform for creating and distributing surveys [7]. This survey included three sets of questions addressing pediatric vision screening attitudes and practices of pediatricians. The first set of questions assessed basic demographics, medical training, and current practice type (academic or private). Next, respondents were asked about comfort with vision screening (5-point Likert scale), prior training on screening, interest in receiving additional training on vision screening, and if they perform vision screening in their practice. If the clinician answered “yes” to performing screening in his or her practice, the survey continued to the second section regarding the frequency of screening, interest in receiving additional training on eye or vision screening, average patient age of first vision screening, and screening method. If the respondent answered “no,” the survey would proceed to the next section for all respondents which asked about the pediatrician’s frequency of encountering patients with ophthalmic concerns and providing ophthalmic referrals.

To obtain responses from pediatricians, the survey was sent through newsletters to members of the Florida Chapter of the American Academy of Pediatrics (FCAAP) and through department emails at Florida universities. The FCAAP is a Florida-based organization representing the interests of pediatricians and pediatric providers in Florida and consists of over 2,700 members [8]. Due to its substantial membership, this organization was chosen as the primary target audience to reach pediatricians in the state. The survey was also sent through pediatric department emails at Florida universities to increase survey reach. After sending the survey as part of the FCAAP newsletter and through pediatric department emails at Florida universities, responses were collected via a voluntary response sample. All methods were approved by the University of Miami Institutional Review Board.

To understand the vision screening practices of the pediatricians surveyed, descriptive statistics of the survey responses were performed using SAS version 9.4 (SAS Institute, Cary, NC), evaluating the demographics of pediatricians surveyed, vision screening attitudes and practices, vision concerns, and ophthalmology referrals from pediatric clinics, and the most common reasons for referrals to an optometrist or ophthalmologist. Additionally, the Jonckheere-Terpstra test, a nonparametric rank-based test for ordinal variables, was used to analyze the trend between decreased comfort with performing pediatric vision screening and decreased rate of urgent referrals to an optometrist or ophthalmologist. Statistical significance was determined at a p-value < 0.05.

## Results

Descriptive statistics from the sample studied are displayed. A total of 46 responses were collected. The majority of pediatricians were female (n=40, 87%), below the age of 45 (n=27, 59%), and White, non-Hispanic (n=24, 52%). Most were attending physicians in pediatrics (n=31, 61%) with no further fellowship training (n=33, 72%, Table 1).

**Table 1:** Demographics of pediatricians surveyed

Total Responses	46 (100%)
Gender	
Female	40 (87%)
Male	6 (13%)
Age	
25-34	19 (41%)
35-44	8 (17%)
45-54	7 (15%)
55-64	7 (15%)
65-74	4 (9%)
75-84	1 (2%)
Ethnicity	
White, non-Hispanic	24 (52%)
Black, non-Hispanic	4 (9%)
Hispanic or Latino	7 (15%)
Asian	7 (15%)
Other	3 (7%)
Prefer not to say	1 (2%)
Profession	
Attending physician in pediatrics	31 (67%)
Resident physician or fellow in pediatrics	9 (20%)
Resident physician or fellow, not pediatrics	1 (2%)
Medical student in pediatrics	1 (2%)
Registered nurse/nurse practitioner in pediatrics	3 (7%)
Other	1 (2%)
Practice	
Academic medicine	39 (85%)
Private practice	6 (13%)
Other	1 (2%)
Region of Florida	
Northeast	2 (4%)
Northwest	1 (2%)
Northcentral	2 (4%)
Southeast	21 (46%)
Southwest	6 (13%)
Central	14 (30%)
Pediatric Fellowship Training	
No fellowship training	33 (72%)
Obtained fellowship training	12 (26%)
Critical Care	1 (2%)
Endocrinology	1 (2%)
Infectious Disease	2 (4%)
Nephrology	3 (7%)
Medical genetics	1 (2%)
Hematology-oncology	2 (4%)
Did not specify training	2 (4%)
No response	1 (2%)

Seventy-eight percent of the 46 respondents (n=36) reported that they perform vision screening in their pediatric clinics. Of respondents who reported performing vision screening procedures, the majority stated that they do so starting from ages zero to two (n=24, 67%). However, only 48% of respondents (n=22) reported feeling “somewhat comfortable” or “extremely comfortable” with performing vision screening. Moreover, only half of respondents reported receiving previous training on performing vision screening (n=23). Thirty-nine percent of clinicians who perform vision screening (n=14) examine for a red reflex and use a visual acuity chart, and an automatic screening device. Automatic screening devices that clinicians most commonly reported using included Welch Allyn Spot (Hill-Rom Holdings, Inc., Chicago, IL) and Plusoptix (Plusoptix, Inc., Kennesaw, GA). Less commonly used screening devices included Ocularphoto (Ocular Technology Inc., Goleta, CA), GoCheck (Gobiquity, Inc., Nashville, TN), and Pediavision (Welch Allyn, Skaneateles Falls, NY) (Table 2).

**Table 2:** Vision screening attitudes and practices of pediatricians surveyed

Total Responses	46 (100%)
Comfort Level with Vision Screenings	
Extremely comfortable	5 (11%)
Somewhat comfortable	17 (37%)
Neither comfortable nor uncomfortable	9 (20%)
Somewhat uncomfortable	10 (22%)
Extremely uncomfortable	3 (7%)
No response	2 (4%)
Prior Training on Vision Screenings	
Yes	23 (50%)
No	23 (50%)
Interest in Receiving Additional Training	
Yes	24 (52%)
Maybe	6 (13%)
No	15 (33%)
No response	1 (2%)
Screening Performed in Clinic	
Yes	36 (78%)
No	10 (22%)
Vision Screening Practices	
Total Number of Clinicians Who Perform Screenings	36 (100%)
Age of First Screening	
0-2 years	24 (67%)
3-5 years	8 (22%)
>5 years	2 (6%)
No response	2 (6%)
Screening Frequency	
Every visit	8 (22%)
Every well child visit	6 (17%)
Every other visit	1 (3%)
Once a year	15 (42%)
Once every 2-3 years	2 (6%)
Other	2 (6%)
No response	2 (6%)
Screening Method	
Only an automatic screening device	1 (3%)
Red reflex and an automatic screening device	7 (19%)
Visual acuity and an automatic screening device	1 (3%)
Only red reflex	1 (3%)
Red reflex and visual acuity	10 (28%)
Red reflex, visual acuity, and an automatic screening device	14 (39%)
No response	2 (6%)

Ophthalmic concerns from parents of patients frequently occurred a few times a month (n=20, 45%) while ophthalmic concerns from patients usually occurred a few times a year (n=23, 52%). All but one respondent had ever made an ophthalmic referral (98%). Forty-two percent of ophthalmic referrals were made a few times a month (n=18), and 51% of clinicians had never provided an urgent referral (n=22, Table 3).

**Table 3:** Vision concerned and ophthalmology referrals in pediatric clinics

Total Responses	44 (100%)
Frequency of Ophthalmic Concerns from Parents	
Every day	1 (2%)
A few times a week	5 (11%)
A few times a month	20 (45%)
A few times a year	15 (34%)
Never	2 (5%)
No response	1 (2%)
Frequency of Ophthalmic Concerns from Patients	
Every day	1 (2%)
A few times a week	4 (9%)
A few times a month	11 (25%)
A few times a year	23 (52%)
Never	4 (9%)
No response	1 (2%)
Hade Ever Made an Ophthalmic Referral	
Yes	43 (98%)
No	1 (2%)
Ophthalmology Referrals in Pediatric Clinics	
Total Number of Clinicians that Had Ever Made a Referral	43 (100%)
Frequency of Ophthalmic Referrals	
Every day	1 (2%)
A few times a week	14 (33%)
A few times a month	18 (42%)
A few times a year	10 (23%)
Previously Made an Urgent Ophthalmology Referral	
Yes	21 (49%)
No	22 (51%)

**Figure 1:**
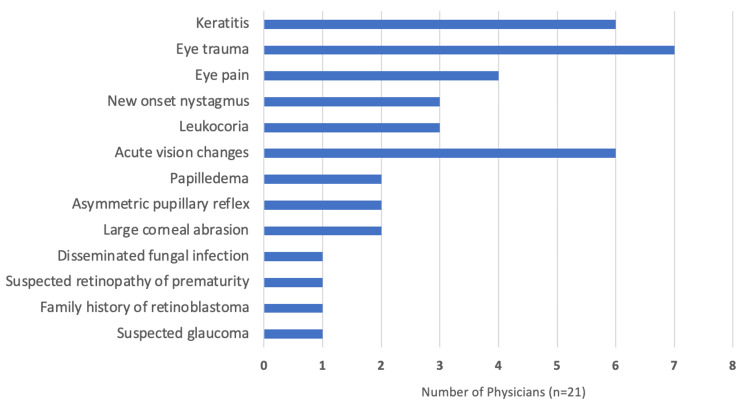
Reasons for urgent ophthalmology referral from pediatricians * Physicians could list more than one reason.

2383The most common reasons for which physicians reported providing an urgent referral were eye trauma, keratitis, and acute vision changes (Figure 1). 

**Figure 2:**
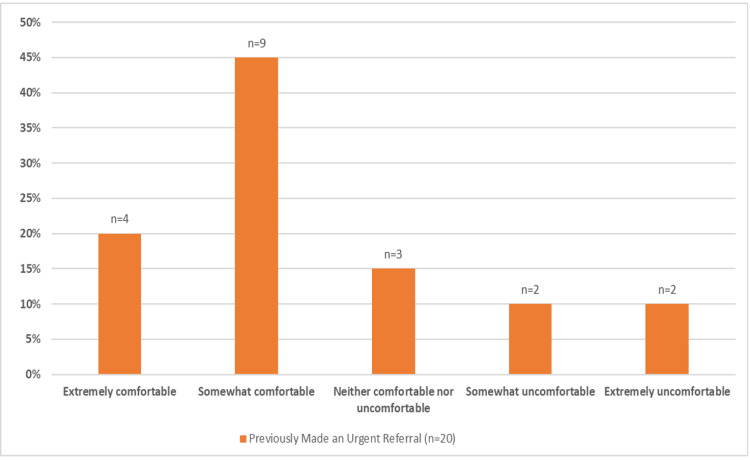
Comfort levels of respondents that previously made an urgent ophthalmology referral (by percent)

The trend between lower levels of comfort with vision screening (from extremely comfortable, somewhat comfortable, neither comfortable nor uncomfortable, somewhat uncomfortable, to extremely uncomfortable) and lower rates of urgent ophthalmic referrals was assessed with the Jonckheere-Terpstra test and was approaching statistical significance (p=0.0705, Table 4). Although the trend was not statistically significant at our alpha, we did observe that 65% (n=13) of urgent referrals were prescribed by respondents who felt somewhat or extremely comfortable providing vision screenings whereas only 20% (n=4) were given by respondents who felt extremely or somewhat uncomfortable with vision screenings (Figure 2).

**Table 4:** Reported comfort with vision screenings and had previously made an urgent ophthalmology referral

Total Responses	42 (100%)	
Comfort Level with Vision Screenings	Has Not Made an Urgent Referral	Made an Urgent Referral
Extremely comfortable	1 (5%)	4 (20%)
Somewhat comfortable	7 (32%)	9 (45%)
Neither comfortable nor uncomfortable	5 (23%)	3 (15%)
Somewhat uncomfortable	8 (36%)	2 (10%)
Extremely uncomfortable	1 (5%)	2 (10%)
Jonckheere–Terpstra test	P-value: 0.0705	

## Discussion

This study examined vision screenings and ophthalmology referrals among a sample of pediatricians in Florida. While most of the pediatricians surveyed began vision screening early with routine vision checks, as recommended by the AAO and AAPOS 2022 joint statement, there was a considerable amount of variability in the way each pediatrician performed his or her vision screening, leading to incongruencies in screening practices. Only 28% (n=10) both assessed for red reflex and used a visual acuity chart, as per minimum guidelines. An additional 39% (n=14) performed both assessments along with the elective use of an automatic vision screening device. Assessing for the red reflex, for instance, is a critical step in catching diseases such as retinoblastoma, which could be life-threatening [9]. Moreover, assessing visual acuity or refraction with an automatic vision screening device is necessary to screen for pediatric conditions such as amblyopia [10]. A similar study assessing preschool vision screening practices nationally in 2006 found that few pediatricians used photoscreening or autorefraction (8%) [11]. One potential explanation for variability in screening practices could be a lack of awareness of screening guidelines. Decreased screening awareness may not only explain the different screening methods but may also account for the lack of comfort reported by many clinicians, as only 48% of respondents (n=22) reported feeling “somewhat comfortable” or “extremely comfortable” with performing vision screening.

This study also found an association between decreased comfort with performing vision screening and decreased urgency in referring patients to an ophthalmologist which was almost statistically significant (p=0.0705). Urgent referrals can be crucial in preventing ophthalmic complications in children. The most common reason for urgent referrals observed in this study was eye trauma, which has been shown to be an important cause of ocular morbidity in children and is the most common cause of preventable blindness in children [12]. High-velocity ocular injury, even with normal visual acuity and eye examination, or chemical injuries are examples of eye trauma that require referral to an ophthalmologist immediately [13,14]. While the present study noted an association between decreased comfort with vision screening and decreased urgency in referrals, it is also important to note that comfort level is only one of many factors that may contribute to whether a pediatrician makes an urgent referral to an ophthalmologist.

Despite reaching a large community of pediatricians in Florida with a survey distribution of over 2,700 individuals, the final sample size was small and limited the analysis. Thus, these results are not applicable to all pediatricians in Florida. It is possible that the observed lack of comfort with vision screening could be a reason that many pediatricians were discouraged from participating in a survey about vision screening practices. If this is the case, individuals with more training on vision screenings may be more forthcoming in sharing their experiences, and increased awareness of the vision screening guidelines put forth by the AAO and AAPOS may help lead to a larger sample size. Delivering surveys through emails rather than in-person measures may have also contributed to the low sample size. Another limitation of this study is that the number of responses varied throughout the study since not all of the respondents completed all the questions of the survey.

Future studies should adopt different methodologies to obtain a larger sample size. Rather than sending surveys via email, which showed low engagement in our study, one may consider other methods to increase survey engagement, such as distributing surveys at a national pediatrics conference or holding focus groups with pediatricians. Although the present study is not generalizable due to the small sample size, we noted an almost significant association between decreased comfort with vision screenings and providing urgent ophthalmic referrals (p=0.0705). Many participants also expressed interest in receiving additional training on vision screenings, with 65% (n=30) saying “yes” or “maybe” to a question about receiving additional training. A future study may consider examining whether increased training on pediatric vision screening procedures may help to improve the consistency of screening and comfort with performing the exam. In a Florida-based study, 29 pediatric or family medicine practices received training on performing vision screening. After four to six months of training, the reported screening frequency of three-year-old children increased, and reported comfort levels with screening three-year-old and four-year-old children were improved, highlighting the importance of vision screening training on increasing vision screening frequency and comfort among PCPs [15].

## Conclusions

Early vision screening is essential to catch signs of childhood vision loss before potentially blinding disease occurs. Understanding the vision screening practices of pediatricians and other PCPs during the years of infancy and early childhood prior to mandated school vision screening can be a crucial part of preventing childhood vision loss. In this survey of pediatricians in the state of Florida, most respondents reported performing early childhood vision screening, but there was notable variability in the way screenings were performed among pediatricians. Moreover, many had never received training on performing the exam or did not feel comfortable performing them. The trend between decreased comfort performing pediatric vision screening and lower rates of urgent eye care referrals was approaching statistical significance (p=0.0705). Future studies should examine whether increased training on vision screenings based on AAPOS and AAO guidelines may help improve consistency of vision screening among pediatricians and improve comfort levels with the exam.
